# Associations of psychological distress with positive psychological variables and activities of daily living among stroke patients: a cross-sectional study

**DOI:** 10.1186/s12888-019-2368-0

**Published:** 2019-12-03

**Authors:** Xiaoxi Wang, Shengjie Shang, Huazhe Yang, Hua Ai, Yin Wang, Shijie Chang, Xianzheng Sha, Lie Wang, Xiran Jiang

**Affiliations:** 1 Department of Sports Medicine, School of Fundamental Sciences, China Medical University, 77 Puhe Road, Shenyang, 110122 People’s Republic of China; 2 Department of Biomedical Engineering, School of Fundamental Sciences, China Medical University, 77 Puhe Road, Shenyang, 110122 People’s Republic of China; 3 Department of Biophysics, School of Fundamental Sciences, China Medical University, 77 Puhe Road, Shenyang, 110122 People’s Republic of China; 4 Department of Social Medicine, School of Public Health, China Medical University, 77 Puhe Road, Shenyang, 110122 People’s Republic of China

**Keywords:** Stroke, Positive psychology, Depression, Anxiety

## Abstract

**Background:**

Depression and anxiety result in psychological distress, which can further affect mental status and quality of life in stroke patients. Exploring the associations between positive psychological variables and symptoms of psychological distress following stroke is of great significance for further psychological interventions.

**Methods:**

A total of 710 stroke patients from the five largest cities in Liaoning Province in China were enrolled into the present study in July 2014. All patients independently completed the questionnaires with respect to psychological distress and positive psychological variables. Depressive and anxiety symptoms were evaluated using Center for Epidemiologic Studies Depression Scale (CES-D) and Self-Rating Anxiety Scale, respectively. Positive psychological variables were evaluated using Perceived Social Support Scale, Adult Hope Scale (AHS), General Perceived Self-Efficacy Scale and Resilience Scale-14 (RS-14). Activities of Daily Living (ADL) was measured using Barthel Index. Factors associated with psychological variables and depressive and anxiety symptoms were identified using *t*-test, ANOVA, correlation and hierarchical linear regression analysis.

**Results:**

Depressive and anxiety symptoms were present in 600 of 710 (84.51%) and 537 of 710 (75.63%) stroke patients enrolled, respectively.

Social support (β = − 0.111, *p* < 0.001) and hope (β = − 0.120, *p* < 0.001) were negatively associated with both depressive and anxiety symptoms.

Resilience (β = − 0.179, *p* < 0.001) was negatively associated with depressive symptoms.

Self-efficacy (β = − 0.135, *p* < 0.001) was negatively associated with anxiety symptoms. Hierarchical regression analyses indicated that ADL accounted for 10.0 and 6.0% of the variance of depressive and anxiety symptoms, respectively.

Social support, resilience, self-efficacy and hope as a whole accounted for 7.5 and 5.3% of the variance of depressive and anxiety symptoms.

**Conclusions:**

The high frequency of depressive and anxiety symptoms among Chinese stroke survivors should receive attentions from all stakeholders. Findings suggested that intervention strategies on ADL, social support, hope, resilience and self-efficacy could be developed to improve psychosocial outcomes for stroke survivors.

## Background

Stroke is a global leading cause of behavioral disabilities and memory loss, and was considered as one of the most common causes of disability among adults. In China, stroke has been pointed to be the leading cause of death and disability-adjusted life-years (DALYs) [1], with the incidence of approximately 1596 per hundred thousand people [2]. The Chinese government has supported developments of China National Stroke Registries and Chinese Stroke Association in 2007 and 2015, respectively, aiming to improve stroke care and outcomes [3]. However, although medical conditions and clinical interventions were improved in recent years, there are still significant gaps between guideline recommendations and clinical practice for stroke survivors. High costs of medical care and insufficient insurance coverage often result in big burden for the whole family [4, 5].

Stroke causes a wide range of physical dysfunctions, resulting in impairments in activities of daily living (ADL), i.e., the ability to complete self-maintenance activities necessary for individuals to be able to live independently. Stroke survivors’ personal ADL level is a key factor in stroke rehabilitation, but that factor is also related to psychological distress [6]. Approximately one-third of stroke patients develop psychological distress very shortly after disease onset [7]. During the stroke treatment process, both the patients and caregivers may experience a high level of psychological distress [8]. Even after the disease is cured, owing maybe due to the high reoccurrence rate and poor prognosis, stroke survivors may still experience high level of psychological distress [9–11]. Researches in this field mainly focused on depression and anxiety in stroke patients [12], since the two symptoms have negative influences on recovery [13] and tend to result in complications and disability or even early death [14, 15]. Thus, reducing depressive and anxiety symptoms of stroke survivors is essential for stroke recovery and extends the survival time of the patients.

Over the past several years, positive psychology has been raising public mental health concerns and receiving increasing attention in the prevention and treatment of depression and anxiety and improvement of quality of life. Both external (social support) and internal factors (hope, resilience and self-efficacy) have been frequently studied in patients with chronic conditions, and have been shown to be helpful in modifying the impact of psychological distress on an individual. In addition, the factors were also pointed to have essential influence on improving patients (e.g., cancer and HIV patients) to cope with stress [16–18]. However, limited research was presented regarding the influence of these factors on the psychological distress of stroke patients. Social support refers to the perceived comfort, caring and assistance that a person receives from his supportive social networks [19]. It has been frequently reported that the level of social support has a strong influence on functional recovery by leading the patients to believe they were concerned and cared for by their friends and families [20, 21]. Previous researches also demonstrated that social support has significant positive effects on psychological health and indicated that social support intervention may help alleviate psychological distress [22–24]. Self-efficacy refers to the strength or extent of one’s belief in one’s own ability to reach goals [16]. Studies have also shown that self-efficacy is a predictor of mood and functional independence for patients with other chronic conditions [25]. Stroke patients who showed greater self-efficacy were reported to be less depressed [26]. Hope is defined as confidence in one’s future. It can provide mental energy through the stages of goal pursuit [27]. During therapy, hope can act as one of the most vital coping style for patients [28]. Resilience is another important positive psychological factor that is considered as a particular trajectory of positive adaptation, helping to protect against psychological distress effectively [29].

The associations of psychological distress with positive psychological variables and ADL following stroke is critical [8, 30]. Although till now, studies in this field using a large sample size are scarce. Nonetheless, there were indications that these factors are strongly related to mood and quality of life of patients with other chronic conditions [16, 31, 32]. Therefore, we conducted the present study with following aims:

(i) Investigating the prevalence of psychological distress including depression and anxiety in stroke patients,
(ii) Identifying the associations of psychological distress with positive psychological variables and ADL among stroke patients

## Methods

### Study design and sample

The cross-sectional study was carried out in July 2014. Participants were recruited by convenience sampling from community hospitals in the five largest cities in Liaoning Province in China. All the participants were informed that their involvement was completely voluntary and that their information would be protected. A total number of 800 stroke patients were enrolled. Seven hundred and forty-one patients returned the questionnaires before the deadline. All participants were all over 18 years of age and were able to read and fully understand both the consent and the questionnaires (Additional file 1). The patients were provided the consent form showing the purpose of the project. The questionnaires were distributed, only after the eligible patients had read and signed the consents by themselves. There were 31 excluded questionnaires because of missing items. A pool of 710 patients (effective response rate: 88.75%) became the final subjects. The project was approved by the Committee on Human Experimentation of China Medical University. Ethics approvals were provided by the same University.

### Demographic characteristics

General Demographic Questionnaires (regarding age, gender, marital status, education level, residence, chronic disease, monthly income and medical payment types) were completed by each patient. Participants were categorized by age into three groups (30–40 years, 41–65 years and 66–90 years), since people ≤40 are obtaining more social resources and job opportunities in China. While, the average retirement age for Chinese citizens is 65. Marital status was categorized as single/separated/divorced/widowed and married/cohabiting. Education levels were categorized as primary school, middle school/high school and college. Residence types were categorized as rural and urban. Monthly income was divided into three groups (≤ 318.8, 318.9–797.0 and ≥ 797.1 US dollars) based on the average income levels in China. Medical payments types were categorized as medical insurance and self-paying.

### Measurement of activities of daily living (ADL)

The Chinese version of Barthel Index has been demonstrated with adequate reliability and validity in Chinese patients [33]. The Barthel Index of Activities of Daily Living [34] was used to measure the patients’ functional disability. Total possible scores range from 0 to 100, with lower scores indicating increased disability. The Cronbach’s alpha for the scale was 0.859 in the current study.

### Measurement of positive psychological variables

#### Social support

The Chinese version of Perceived Social Support scale (PSSS) [35] containing 12 items was used in this study. The scale consists of supports from family and friends. Each item was scored on a 7-point likert scale ranging from 1 (very strongly disagree) to 7 (very strongly agree), with a higher total score meaning a higher level of social support. The Chinese PSSS has been used among Chinese patients and showed good reliability [36]. The Cronbach’s alpha of the scale was calculated to be 0.962 in this research.

#### Hope

The Chinese version of Adult Hope Scale (AHS) [37] was used as the measurement tool to assess levels of hope in stroke patients. The scale contains 8 items and 4 fillers. Each item was scored on a 4-point likert scale ranging from 1 to 4. A higher total score reflects a higher hope level. The Chinese version of AHS is considered to have adequate reliability when used with Chinese patients [38]. The Cronbach’s alpha of the scale was 0.841 in the present study.

#### Resilience

The Chinese version of Wagnild and Young Resilience Scale-14 (RS-14) [39] was used to measure the patients’ resilience. Each item was rated on a 7-point scale ranging from 1 (strongly disagree) to 7 (strongly agree), with a higher total score reflecting a higher level of resilience. The Chinese resilience scale has been used among Chinese patients and showed good reliability [40]. The Cronbach’s alpha of the scale was 0.956 in this study.

#### Self-efficacy

The Chinese version of general perceived self-efficacy scale [41] was used to assess patients’ self-efficacy. The scale consists of 10 items rated on a 4-point scale, ranging from 1 (not at all true) to 4 (exactly true), with a higher total score indicating a higher self-efficacy level. The Chinese version of the scale has been used in Chinese patients with good reliability [42]. The Cronbach’s alpha for the scale was 0.897 in the current study.

### Measurement of depression and anxiety

#### Depressive symptom

Depressive symptoms were measured using the Chinese version of the Center for Epidemiologic Studies Depression Scale (CES-D) [43], which consists of 20 items. Each item has four response categories ranging from 0 (almost never) to 3 (always). Participants that have a scoring 16 or more were believed to have depressive symptoms [44, 45]. The Chinese CES-D has demonstrated adequate reliability in Chinese people in previous reports [46, 47]. The Cronbach’s alpha for the scale was 0.908 in the current study.

#### Anxiety symptom

The Chinese version of Zung self-rating anxiety scale (SAS) [48] was used to assess the anxiety symptoms in stroke survivors. The scale contains 20 items ranging from 0 (almost never) to 3 (always). To standardize the total score, the raw score was multiplied with 1.25, then the integer part is used as the standard total score. The presence of anxiety symptom was defined as the standardized score ≥ 50 [49]. The Chinese version of the scale has been used in Chinese patients and showed adequate reliability in our previous studies [49]. The Cronbach’s alpha for the scale was 0.886 in the present study.

### Statistical analysis

All analyses were performed using Statistical Product and Service Solutions software for Windows version 17.0 (SPSS, Inc., Chicago, IL). The level of statistical significance (*p* value) was set at 0.05 (two-tailed). Descriptive statistics of the demographic characteristics were conducted with mean, standard deviation (SD), number (N) and percentage (%) as appropriate. T-test and one-way analysis of variance (ANOVA) were used to compare differences in categorical variables. Pearson’s correlation was used to examine correlations among ADL, psychological variables, depression and anxiety. In step 1, demographic characteristics were added. The activities of daily living (ADL) was entered into step 2. Ultimately, positive psychological variables were added in step 3. Depression and anxiety were included as dependent variables in the hierarchical linear regressions, respectively. Hierarchical linear regressions were used to explore the effects of demographics factors, ADL, social support, hope, resilience, self-efficacy on depression and anxiety symptoms, as well as the interaction effects among the predictors. Standardization regression coefficient (β), F, R^2^ and R^2^ changes(△R^2^) for each step were calculated. All variables were standardized before the hierarchical regression analysis.

## Results

### Descriptive statistics

Descriptive statistics (*n* = 710, 58.2% male) was conducted and showed the results in Table 1. The participants were aged 30–90 years (mean ± SD = 61.67 ± 11.97). Five hundred and fifty-three (75.1%) patients were married. One hundred and thirteen (15.9%) patients received college or higher education. While, one hundred and seventy-two (24.2) patients received primary school or lower education. Four hundred and twenty-nine (60.4%) patients were living in an urban area. Six hundred and thirty-three (89.2%) patients had at least one chronic condition except stroke. One hundred and sixty-nine (23.8%) patients had a monthly income level of ≤318.8 US dollars, with fifty-eight (8.2%) ≥ 797.1. For medical payment types, five hundred and eighty-six (82.5%) patients had medical insurance, the rest of which were paying the medical fees by themselves.

**Table 1:** Descriptive statistics of demographic characteristics of stroke survivors (*n* = 710)

					Mean ± SD		
	n (%)	Depression	Anxiety	Social Support	Hope	Resilience	Self- efficacy
*Age* (years)							
= < 40	38 (5.4)	22.47 ± 9.47	53.03 ± 11.55	65.66 ± 15.15	24.13 ± 4.55	64.97 ± 18.20	28.61 ± 7.55
41–65	381 (53.7)	23.72 ± 9.36	55.59 ± 10.71	59.82 ± 13.71	21.90 ± 3.76	64.71 ± 16.42	25.59 ± 5.61
> = 66	291 (41.0)	25.30 ± 8.92	57.05 ± 11.42	59.62 ± 13.06	21.58 ± 3.51	63.63 ± 14.65	24.50 ± 4.69
*F*		3.225^*^	2.958	3.465*	7.926**	0.423	10.945**
*Gender*							
Male	413 (58.2)	24.77 ± 9.41	56.37 ± 10.91	59.11 ± 14.56	21.90 ± 3.84	62.86 ± 15.86	25.59 ± 5.46
Female	297 (41.8)	23.66 ± 8.92	55.61 ± 11.32	61.35 ± 11.98	21.87 ± 3.61	66.25 ± 15.54	24.92 ± 5.42
*T*		1.571	0.901	2.243*	0.105	2.828**	1.623
*Marital status*							
Married	533 (75.1)	24.05 ± 9.51	56.02 ± 11.17	60.90 ± 12.91	22.01 ± 3.77	64.78 ± 15.80	25.32 ± 5.48
Single/Divorced/Widow	177 (24.9)	25.08 ± 8.26	56.13 ± 10.85	57.50 ± 15.17	21.51 ± 3.66	62.76 ± 15.78	25.25 ± 5.36
*T*		1.300	0.108	2.671**	1.547	1.475	0.149
*Education level*							
Primary school	172 (24.2)	24.91 ± 10.02	56.70 ± 11.92	59.20 ± 12.13	20.82 ± 3.67	62.15 ± 14.13	24.09 ± 5.33
Middle school	425 (59.9)	24.71 ± 8.75	56.83 ± 10.50	59.64 ± 13.62	21.81 ± 3.43	64.42 ± 15.79	25.05 ± 4.74
College	113 (15.9)	21.84 ± 9.36	52.13 ± 11.15	62.88 ± 15.17	23.78 ± 4.27	67.01 ± 17.86	28.12 ± 7.02
*F*		4.877**	8.579**	3.003	22.725**	3.288*	20.958**
*Residence types*							
Urban	429 (60.4)	23.82 ± 9.16	54.99 ± 10.99	60.79 ± 14.14	22.41 ± 3.90	64.90 ± 16.01	26.27 ± 5.58
Rural	281 (39.6)	25.04 ± 9.28	57.67 ± 11.04	58.92 ± 12.61	21.09 ± 3.35	63.34 ± 15.48	23.84 ± 4.89
*T*		1.720	3.177**	1.794	4.648**	1.281	5.940**
*Chronic disease*							
No	77 (10.8)	21.00 ± 10.31	51.85 ± 12.57	63.93 ± 16.02	24.27 ± 4.50	66.03 ± 17.59	29.53 ± 6.22
Yes	633 (89.2)	24.71 ± 9.00	56.56 ± 10.79	59.58 ± 13.19	21.60 ± 3.54	64.07 ± 15.58	24.79 ± 5.12
*T*		3.354**	3.550**	2.667*	6.061**	0.933	7.474**
*Monthly income* (US dollars)							
<=381.8	169 (23.8)	22.32 ± 10.31	54.53 ± 11.10	60.94 ± 11.65	20.33 ± 3.61	63.61 ± 13.68	23.73 ± 5.43
318.9–797.0	483 (68.0)	24.73 ± 8.52	56.45 ± 10.70	59.15 ± 14.26	22.46 ± 3.66	64.18 ± 16.57	25.93 ± 5.37
> = 797.1	58 (8.2)	26.52 ± 10.54	57.11 ± 13.64	64.98 ± 11.76	21.69 ± 3.60	67.07 ± 15.00	24.74 ± 5.27
*F*		6.195**	2.174	5.325**	21.441**	1.064	10.815**
*Medical payments types*							
Insurance	586 (82.5)	24.04 ± 9.25	55.84 ± 11.12	61.54 ± 13.73	22.06 ± 3.83	65.35 ± 16.32	25.70 ± 5.59
Self-Pay	124 (17.5)	25.56 ± 8.99	57.07 ± 10.87	53.02 ± 10.27	21.06 ± 3.22	59.24 ± 11.93	23.44 ± 4.27
*T*		1.677	1.126	7.861**	2.723**	4.822**	5.043**

* *p* < 0.05. ** *p* < 0.01

Independent sample *t*-test or one-way ANOVA results showed: (i) Patient age, education level, chronic disease and monthly income were significantly related to depressive symptoms. (ii) Patient education level, residence types and chronic disease were significantly related to anxiety. (iii) Patients age, gender, marital status, chronic disease, monthly income and medical payments types were significantly related to social support. (iv) Patients age, education level, residence types, chronic disease, monthly income and medical payments types were significantly related to hope. (v) Patients gender, education level and medical payments types were significantly related to resilience. (vi) Patients age, education level, residence types, chronic disease, monthly income and medical payments types were significantly related to self-efficacy.

The levels of depressive and anxiety symptoms, age, activities of daily living, social support, hope, resilience and self-efficacy, were listed in Table 2. The mean score of depressive and anxiety symptoms were 24.30 and 56.05, respectively. The overall prevalence of depressive and anxiety symptoms was 84.51% (600 of 710 patients) and 75.63% (537 of 710 patients), respectively.

**Table 2:** Descriptive statistics for continuous variables

Variables	Mean	SD	n (%)
Depressive symptoms	24.30	9.22	
Score ≥ 16			600 (84.51)
Anxiety symptoms	56.05	11.08	
Score ≥ 50			537 (75.63)
Age (years)	61.67	11.97	
Activities of Daily Living	67.69	14.37	
Social support	60.05	13.58	
Hope	21.89	3.75	
Resilience	64.28	15.81	
Self-efficacy	25.31	5.45	

### Correlations among ADL, psychological variables, depression and anxiety

Pearson’s correlation coefficients among ADL, social support, hope, resilience, self-efficacy, depressive and anxiety symptoms were shown in Table 3. The depressive and anxiety symptoms were significantly negatively correlated with all the psychological variables. The ADL was correlated with hope, resilience, self-efficacy, depression and anxiety. Patient’s age showed no correlation with social support or resilience.

**Table 3:** Means, standard deviations and correlations among ADL, psychological variables, depression and anxiety

		1	2	3	4	5	6	7	8
1	Age	1							
2	Activities of Daily Living	−0.163**	1						
3	Social support	0.060	0.072	1					
4	Hope	−0.113**	− 0.343**	0.224**	1				
5	Resilience	−0.033	− 0.236**	0.534**	0.250**	1			
6	Self-efficacy	−0.160**	− 0.356**	0.218**	0.533**	0.342**	1		
7	Depression	0.095*	−0.343**	− 0.258**	− 0.245**	− 0.322**	− 0.210**	1	
8	Anxiety	0.089*	−0.285**	− 0.223**	− 0.216**	− 0.210**	− 0.111**	−0.821**	1

* *p* < 0.05. ** *p* < 0.01 (two-tailed)

### Hierarchical regression analyses

Table 4 shows the hierarchical regression analyses of ADL, social support, hope, resilience, self-efficacy on depression and anxiety symptoms. Patients’ age, gender, marital status, education level, residence types, chronic disease, monthly income and medical payment types were entered into step 1 to work as control variables, since these factors were significantly related to depression and anxiety as revealed by t-test and ANOVA (See Table 1). Depressive and anxiety symptoms were used as dependent variables. For depression model, social support, hope and resilience showed significantly negative impact on depressive symptoms. As shown in the last row of Table 4, positive psychological variables as a whole accounted for 7.5% of the variance. ADL was found to have a significant and negative impact on depressive symptoms, and accounted for 10.0% of the variance. For anxiety model, social support, hope and self-efficacy showed significantly negative effects. Positive psychological variables as a whole accounted for 5.3% of the variance. ADL accounted for 6.0% of the variance.

**Table 4:** Hierarchical regression analyses of stroke survivors

	Depression			Anxiety		
	Step 1 (β)	Step 2 (β)	Step 3 (β)	Step 1 (β)	Step 2 (β)	Step 3 (β)
*Step 1 Demographic Characteristics*						
Age	0.039	0.013	0.019	0.028	0.008	0.016
Gender	−0.055	− 0.057	− 0.030	− 0.031	− 0.032	− 0.009
Marital status	0.062	0.049	0.021	0.019	0.009	−0.019
Education_1	−0.048	−0.021	− 0.007	−0.020	0.000	0.010
Education_2	−0.124*	−0.075	− 0.041	−0.132**	− 0.094	−0.070
Residence types	0.067	0.037	0.035	0.106**	0.082*	0.089*
Chronic disease	0.094*	0.049	0.031	0.085*	0.049	0.042
Monthly income_1	0.184**	0.218	0.216**	0.142**	0.168**	0.165**
Monthly income_2	0.179**	0.184	0.195**	0.117**	0.121**	0.136**
Medical payment types	0.074*	0.051	0.000	0.040	0.022	−0.014
*Step 2 Activities of Daily Living (ADL)*						
		−0.335**	−0.271**		−0.261**	− 0.237**
*Step 3 Positive psychological variables*						
Social support			−0.111**			−0.142**
Hope			−0.120**			−0.148**
Resilience			−0.179**			−0.083
Self-efficacy			−0.031			−0.135**
F	4.704**	11.806**	14.009**	4.213**	8.114**	9.233**
R^2^	0.069	0.169	0.244	0.062	0.123	0.176
Adjusted R^2^	0.054	0.155	0.227	0.047	0.107	0.157
△R^2^	0.069	0.100	0.075	0.062	0.060	0.053

*Notes*. Education_1 means Middle school vs. Primary school. Education_2 means College vs. Primary school. Monthly income_1 means ‘318.9–797.0 (US dollars)’ vs. ‘< 318.8’. Monthly income_2 means ‘> 797.1 (US dollars)’ vs. ‘< 318.8’

* *p* < 0.05. ** *p* < 0.01 (two-tailed)

## Discussion

### Prevalence of psychological distress in stroke patients

This study represented investigation of the prevalence of depressive and anxiety symptoms, and first explored the concurrent effects of positive psychological variables on psychological distress among stroke survivors using a large sample size. Our results showed that the overall prevalence of depressive and anxiety symptoms was 84.51% (600 of 710 stroke patients) and 75.63% (537 of 710 stroke patients), respectively, which were higher compared with previous studies [50]. We assume the limited medical resources, relatively poor medical conditions and complex family relationships in China might be possible reasons to explain why the Chinese stroke patients are generally experiencing both depression and anxiety disorder.

### Effects of demographic characteristics on psychological distress

Both monthly income and medical payment types were shown to have significant impacts on depressive and anxiety symptoms. The stroke survivors having a relatively higher income or sufficient medical care can afford the payments for treatments and recovery process and have lower psychological distress. This might be explained in part by the fact that stroke has a long-term financial impact, which makes the patients moods to be partly affected by their financial capabilities. Our findings revealed that education levels were negatively correlated with psychological distress. Patients with higher education levels (middle school vs. primary school and college vs. primary school) always showed lower levels of depressive and anxiety symptoms. This was in accordance with a previous research on cancer patients [49]. We assume that patients with higher education level trend to obtain better job, income and higher level of social status, which can help them with stronger self-adjustment capacity in dealing with psychological distress [31]. The residence type was critical on the levels of medical treatments that can be received by the stroke patients in China. We found that the residence type was correlated with anxiety. This may be explained by the fact that urban and rural differences in medical conditions in China. Patients living in rural can receive less medical care compared with those living in urban, and thus experience more anxiety. The residence type did not appear to influence the prevalence of depressive symptom. This was consisted with our previous study on Chinese people [51]. We noticed that previous studies have revealed the symptomatic distinctions between depressive and anxiety symptoms [16, 52]. Generally, the depressive symptoms mainly included sadness, hopelessness and anhedonia. While, anxiety symptoms mainly included worry, fear and insomnia. This may explain the potential differences of psychological factors between depression and anxiety [52].

### Effects of ADL and positive psychological variables on psychological distress

In addition to the effect of the demographic characteristics, ADL was shown to be significantly and inversely correlated with both depressive and anxiety symptoms. Stroke has an immense impact on physical functioning. Given the importance that daily activities are necessary for the stroke patients to live independently (e.g. eating, toileting and moving) [53], the patients with higher level of ADL would require less support from others and have better psychological statues. Our findings revealed that the ADL accounted for 10.0 and 6.0% of the variances in depressive and anxiety symptoms, respectively, indicating that physical function has a big impact on the patient’s psychological status. Previous studies have revealed that positive psychological resources have considerable impact on chronic patients (e.g. cancer) and are remarkably important for one’s ability to recover from the current difficulties and to facilitate psychological well-being [32, 48, 54, 55]. In this study, social support, hope, resilience and self-efficacy were shown to be negatively correlated with depressive and anxiety symptoms in stroke survivors. The four variables as a whole accounted for 7.5 and 5.3% of the variance in depressive and anxiety symptoms, respectively.

Social support occurs in interpersonal relationships and is important to an individual’s ability to cope with negative events [56]. To stroke survivors, social support has been recognized as an important determinant of psychological well-being [57]. High levels of social support were believed to be associated with more extensive recovery of functional capacity and can give better outcome for stroke patients [58]. Previous studies showed that social support is most beneficial to stroke patients with greater functional disability [59]. In this study, social support also showed very significant impacts on both depressive and anxiety symptoms. Considering the limited medical resources in China, once the stroke patients return home after hospitalization, they completely rely on physical and emotional support from their families and friends to live. Thus, social support would be very important and can act to prevent the onset of distress for stroke patients. Psychological resilience comprises a range of psychological, social and environmental factors. It is an important capability of an individual bouncing back in the face of setbacks, such as coping with a long term condition, and return to a previous state of normal functioning [60, 61]. In this study, resilience was found to be significantly associated with depressive symptoms, which was consistent with previous studies showing resilience has considerable impacts in adults with chronic disabilities [62, 63] and also associated with improved psychosocial outcomes in older people [64, 65]. Our results showed that the resilience did not correlate with anxiety in stroke patients. In our previous research on Chinese cancer patients, resilience was also found to be associated with depression, but not with anxiety [16]. Self-efficacy was introduced by Bandura (1997), being recognized as an important part of mental health [66]. In this study, significant relation was found between self-efficacy and anxiety, but not between self-efficacy and depression among stroke patients. The result was inconsistent with a previous report, which showed that stroke patients with a lower self-efficacy were more depressed [26]. However, as revealed by a previous study that analyzed symptomatic distinctions between the two disorders [52], the anxiety symptoms mainly included worry and fear. And in some cases, self-efficacy was not significantly associated with psychological distress, although the factor was believed to be an essential trait in predicting depression [67]. Hope is another important internal positive psychological construct and was believed to contribute to the prediction of functional outcomes in rehabilitation populations [68]. Our results indicated that hope has significant impacts on both depressive and anxiety symptoms in stroke patients. The result was consistent with the previous research that hope was shown to play an important role in assisting stroke patients [69].

### Implication

It was demonstrated that psychological distress was frequently associated with poor quality of life [12]. Our results suggested that early interventions on stroke patients’ social support, hope, resilience and self-efficacy traits would alleviate the patients’ distress symptoms, and would possibly improve their quality of life. Thus, positive psychology intervention would be a potential target for practical intervention in stroke survivors. In addition to the practical implications, this study has also evaluated the prevalence of psychological distress among stroke patients, which would be helpful for early identification of the psychological disorders that could be reduced through further interventions. Early distress screening for patients may also improve clinician-patient communication to enhance health outcomes [70]. Although the positive psychological resources plus ADL as a whole accounted for 17.5 and 11.3% of the variances in depressive and anxiety symptoms, respectively, there was still a large proportion of variance remained unexplained.

### Limitations

There were several limitations in this study. First, since both the psychological variables and distress symptoms were measured simultaneously, we are unable to draw causal conclusions. The current findings are suggested to be further confirmed by a longitudinal study in the future. Second, considering all the self-report data were obtained from questionnaires, bias might be introduced. The patients may have overestimate or underestimate the relationships between the psychological factors and distress. Third, seeing that the participants were recruited by convenience sampling, limited representations might be obtained. Further studies using other types of sampling methods which may produce a more representative sample [71, 72] are needed. In the future, a multicenter study covering more provinces would also be conducted to ensure a representative distribution of the population. Furthermore, a control group who are not suffering from stroke was lacking, as a result, we are not certain if the findings in the study were typical for stroke patients or would also be found in the general population. In order to obtain general conclusions, further studies with control population in parallel are considered.

## Conclusions

In this study, very high frequency of depressive (84.51%) and anxiety symptoms (75.63%) among Chinese stroke survivors were detected. Positive psychological resources as a whole accounted for 7.5 and 5.3% of the variance in depressive and anxiety symptoms, respectively. Social support and hope were significantly and negatively associated with both depressive and anxiety symptoms. Resilience was negatively associated with depressive symptoms. While, self-efficacy was negatively associated with anxiety symptoms. Our results also revealed that the ADL had significant impact on the psychological status of stroke patients. The findings from this study have implications for the development of intervention strategies to promote positive psychological variables of social support, hope, resilience and efficacy to improve psychosocial outcomes for stroke survivors. Stroke survivor’s distress symptoms could be alleviated by fostering the positive psychological capitals.

## Supplementary information

**Additional file 1.** Mental health questionnaire for stroke patients.

## Data Availability

The datasets used and/or analyzed during the current study are available from the corresponding author on reasonable request.

## Abbreviations

CES-D: Center for Epidemiologic Studies Depression Scale
SAS: Zung self-rating anxiety scale;
AHS: Adult Hope Scale
RS-14: Resilience Scale-14
ADL: Activities of Daily Living
SPSS: Statistical Package for the Social Sciences
ANOVA: Analysis of variance.

## References

[1] Zhou MG, Wang HD, Zeng XY, Yin P, Zhu J, Chen WQ (2019). Mortality, morbidity, and risk factors in China and its provinces, 1990–2017: a systematic analysis for the Global Burden of Disease Study 2017. Lancet..

[2] Wang W, Jiang B, Sun H, Ru X, Sun D, Wang L (2017). Prevalence, incidence, and mortality of stroke in China: results from a nationwide population-based survey of 480 687 adults. Circulation..

[3] Wang YL, Li ZX, Zhao XQ, Wang D, Li H, Xian Y (2017). Stroke care quality in China: substantial improvement, and a huge challenge and opportunity. Int J Stroke.

[4] Li Z, Wang C, Zhao X, Liu L, Wang C, Li H (2016). Substantial progress yet significant opportunity for improvement in stroke care in China. Stroke..

[5] Liu LP, Wand D, Lawrence Wong KS, Wang YJ (2011). Stroke and stroke care in China huge burden, significant workload, and a national priority. Stroke..

[6] Bakken LN, Kim HS, Finset A, Lerdal A (2012). Stroke patients’ functions in personal activities of daily living in relation to sleep and socio-demographic and clinical variables in the acute phase after first-time stroke and at six months of follow-up. J Clin Nurs.

[7] Hackett ML, Pickles K (2014). Part I: frequency of depression after stroke: an updated systematic review and meta-analysis of observational studies. Int J Stroke.

[8] Jones F, Partridge C, Reid F (2008). The stroke self-efficacy questionnaire: measuring individual confidence in functional performance after stroke. J Clin Nurs.

[9] Carod-Artal FJ, Egido JA (2009). Quality of life after stroke: the importance of a good recovery. Cerebrovasc Dis.

[10] Bonita R, Mendis S, Truelsen T, Bogousslavsky J, Toole J, Yatsy F (2004). The global stroke initiative. Lancet Neurol.

[11] Braamse AM, Van Meijel B, Visser O, Van Oppen P, Boenink AD, Eeltink C (2010). Distress and quality of life after autologous stem cell transplantation: a randomized clinical trial to evaluate the outcome of a web-based stepped care intervention. BMC Cancer.

[12] Van Mierlo ML, Schröder C, Van Heugten CM, Post MW, De Kort PL, Visser-Meily JM (2014). The influence of psychological factors on health-related quality of life after stroke: a systematic review. Int J Stroke.

[13] Prieto JM, Atala J, Blanch J, Carreras E, Rovira M, Cirera E (2005). Role of depression as a predictor of mortality among cancer patients after stem-cell transplantation. J Clin Oncol.

[14] Yang YL, Sui GY, Liu GC, Huang DS, Wang SM, Wang L (2014). The effects of psychological interventions on depression and anxiety among Chinese adults with cancer: a meta-analysis of randomized controlled studies. BMC Cancer.

[15] Ryan H, Schofield P, Cockburn J, Butow P, Tattersall M, Turner J (2005). How to recognize and manage psychological distress in cancer patients. Eur J Cancer Care.

[16] Wang ZY, Liu L, Shi M, Wang L (2016). Exploring correlations between positive psychological resources and symptoms of psychological distress among hematological cancer patients: a cross-sectional study. Psychol Health Med.

[17] Sun W, Wu M, Qu P, Lu C, Wang L (2014). Psychological well-being of people living with HIV/AIDS under the new epidemic characteristics in China and the risk factors: a population-based study. Int J Infect Dis.

[18] Sui GY, Hu S, Sun W, Wang Y, Liu L, Yang XS (2014). Prevalence and associated factors of depressive symptoms among Chinese male correctional officers. Int Arch Occup Environ Health.

[19] Wallston BS, Alagna SW, DeVellis BM, DeVellis RF (1983). Social support and physical health. Health Psychol.

[20] Ikeda A, Kawachi I, Iso H, Iwasaki M, Inoue M, Tsugane S (2013). Social support and cancer incidence and mortality: the JPHC study cohort II. Cancer Causes Control.

[21] Dreyer J, Schwartz-Attias I (2014). Nursing care for adolescents and young adults with cancer: literature review. Acta Haematol.

[22] Liu L, Xu NL, Wang L (2017). Moderating role of self-efficacy on the associations of social support with depressive and anxiety symptoms in Chinese patients with rheumatoid arthritis. Neuropsychiatr Dis Treat.

[23] Zhang H, Qian HZ, Meng SQ, Shu M, Gao YZ, Xu Y (2015). Psychological distress, social support and medication adherence in patients with ischemic stroke in the mainland of China. J Huazhong Univ Sci Technol.

[24] Cumming TB, Cadilhac DA, Rubin G, Crafti N, Pearce DC (2008). Psychological distress and social support in informal caregivers of stroke survivors. Brain Impair.

[25] Barry L, Guo Z, Kerns RD, Duong BD, Reid CM (2003). Functional self-efficacy and pain-related disability among older veterans with chronic pain in the primary care setting. Pain..

[26] Robinson-Smith G, Johnston MV, Allen J (2000). Self-care self-efficacy, quality of life, and depression after stroke. Arch Phys Med Rehabil.

[27] Snyder CR (2002). Hope theory: rainbows in the mind. Psychol Inq.

[28] Ebright PR, Lyon B (2002). Understanding hope and factors that enhance hope in women with breast cancer. Oncol Nurs Forum.

[29] Mancini AD, Bonanno GA (2009). Predictors and parameters of resilience to loss: toward an individual differences model. J Pers.

[30] Hellstrom K, Lindmark B, Wahlberg B, Fugl-Meyer AR (2003). Self-efficacy in relation to impairments and activities of daily living in elderly patients with stroke: a prospective investigation. J Rehabil Med.

[31] Li MY, Yang YL, Wang L (2016). Effects of social support, hope and resilience on quality of life among Chinese bladder cancer patients: a cross-sectional study. Health Qual Life Outcomes.

[32] Wang Y, Bao Y, Liu L, Ramos A, Wang Y, Wang L (2015). The mediating effect of self-efficacy in the relationship between social support and post-traumatic stress disorder symptoms among patients with central system tumors in China: a cross-sectional study. Psycho-oncol..

[33] Zhang L, Lou P, Zhu Y, Chen P, Zhang P, Yu J (2013). Impact of risk factors, activities and psychological disorders on the health of patients with chronic obstructive pulmonary disease in China: a cross-sectional study. BMC Public Health.

[34] Mahoney FI, Barthel DW (1965). Functional evaluation: the barthel index. Md State Med J.

[35] Zimet GD, Dahlem NW, Zimet SG, Farley GK (1988). The multidimensional scale of perceived social support. J Pers Assess.

[36] Wang Y, Zhu X, Yang Y, Yi J, Tang L, He J (2015). What factors are predictive of benefit finding in women treated for non-metastatic breast cancer? A prospective study. Psycho-oncol..

[37] Snyder CR, Harris C, Anderson JR, Holleran SA, Irving LM, Sigmon ST (1991). The will and the ways: development and validation of an individual-differences measure of hope. J Pers Soc Psychol.

[38] Chen LL, Peng L, Tang T, Li M (2012). Correlation between mental health and coping style in gynecological cancer patients (in Chinese). J Third Mil Med Univ.

[39] Wagnild GM (2009). The resilience scale user’s guide for the US English version of the resilience scale and the 14-item resilience scale (RS-14). The Resilience Center.

[40] Tian J, Hong JS (2013). Validation of the Chinese version of the resilience scale and its cutoff score for detecting low resilience in Chinese cancer patients. Support Care Cancer.

[41] Schwarzer R, Jerusalem M, Weinman J, Wright S, Johnston M, Weinman J, Wright S, Johnston M (1995). Generalized Self-Efficacy scale. Measures in health psychology: A user’s portfolio. Causal and control beliefs. Windsor: NFER-NELSON.

[42] Chiu FP, Tsang HW (2004). Validation of the Chinese general self-efficacy scale among individuals with schizophrenia in Hong Kong. Int J Rehabil Res.

[43] Hann D, Winter K, Jacobsen P (1999). Measurement of depressive symptoms in cancer patients: evaluation of the center for epidemiological studies depression scale (CES-D). J Psychosom Res.

[44] Zhang J, Lester D, Zhao S, Zhou C (2013). Suicidal ideation and its correlates: testing the interpersonal theory of suicide in Chinese students. Arch Suicide Res.

[45] Song Y, Huang Y, Liu D, Kwan JS, Zhang F, Sham PC (2008). Depression in college: depressive symptoms and personality factors in Beijing and Hong Kong college freshmen. Compr Psychiatry.

[46] Wang JN, Sun W, Chi TS, Wu H, Wang L (2010). Prevalence and associated factors of depressive symptoms among Chinese doctors: a cross-sectional survey. Int Arch Occup Environ Health.

[47] Wang Y, Yao LT, Liu L, Yang XS, Wu H, Wang J (2014). The mediating role of self-efficacy in the relationship between big five personality and depressive symptoms among Chinese unemployed population: a cross-sectional study. BMC Psychiatry.

[48] Zung WW (1971). A rating instrument for anxiety disorders. Psychosomatics..

[49] Li M, Wang L (2016). The associations of psychological stress with depressive and anxiety symptoms among Chinese bladder and renal cancer patients: the mediating role of resilience. PLoS One.

[50] Ayerbe L, Ayis S, Crichton S, Wolfe CD, Rudd AR (2014). The long-term outcomes of depression up to 10 years after stroke; the South London stroke register. J Neurol Neurosur Ps.

[51] Shi M, Liu L, Wang ZY, Wang L (2016). Prevalence of depressive symptoms and its correlations with positive psychological variables among Chinese medical students: an exploratory cross-sectional study. BMC Psychiatry.

[52] Aina Y, Susman JL (2006). Understanding comorbidity with depression and anxiety disorders. J Am Osteopath Assoc.

[53] Bozo O, Guarnaccia CA (2010). Activities of daily living, social support, and future health of older americans. Aust J Psychol.

[54] Li Y, Yang Y, Zhang R, Yao K, Liu Z (2015). The mediating role of mental adjustment in the relationship between perceived stress and depressive symptoms in hematological cancer patients: a cross-sectional study. PLoS One.

[55] Lu L, Pan B, Sun W, Cheng L, Chi T, Wang L (2010). Quality of life and related factors among cancer caregivers in China. Psychiatry Clin Neurosci.

[56] Knapp P, Hewison J (1998). The protective effects of social support against mood disorder after stroke. Psychol Health Med.

[57] Kruithof WJ, Van Mierlo ML, Visser-Meily JM, Van Heugten CM, Post MW (2013). Associations between social support and stroke survivors’ health-related quality of life-a systematic review. Patient Educ Couns.

[58] Glass TA, Matchar BD, Belyea M, Feussner JR (1993). Impact of social support on outcome in first stroke. Stroke..

[59] Wilcox VL, Kasl SV, Berkman LF (1994). Social support and physical disability in older people after hospitalization: a prospective study. Health Psychol.

[60] Tugade MM, Fredrickson BL (2004). Resilient individuals use positive emotions to bounce back from negative emotional experiences. J Pers Soc Psychol.

[61] He F, Cao R, Feng Z, Guan H, Peng J (2013). The impacts of dispositional optimism and psychological resilience on the subjective well-being of burn patients: a structural equation modelling analysis. PLoS One.

[62] King G, Cathers T, Brown E, Specht JA, Willoughby C, Polgar JM (2003). Turning points and protective processes in the lives of people with chronic disabilities. Qual Health Res.

[63] Harris PB (2008). Another wrinkle in the debate about successful aging: the undervalued concept of resilience and the lived experience of dementia. Int J Aging Hum Dev.

[64] Windle G, Bennett KM, Noyes J (2011). A methodological review of resilience measurement scales. Health Qual Life Outcomes.

[65] Hardy SE, Concato J, Gill TM (2004). Resilience of community-dwelling older persons. J Am Geriatr Soc.

[66] Jones F, Riazi A (2011). Self-efficacy and self-management after stroke: a systematic review. Disabil Rehabil.

[67] Karademas EC (2006). Self-efficacy, social support and well-being: the mediating role of optimism. Personal Individ Differ.

[68] Kortte KB, Stevenson JE, Hosey MM, Castillo R, Wegener ST (2012). Hope predicts positive functional role outcomes in acute rehabilitation populations. Rehabil Psychol.

[69] Weis R, Speridakos EC (2011). A meta-analysis of hope enhancement strategies in clinical and community settings. Psychology of Well-Being.

[70] Carlson LE, Waller A, Mitchell AJ (2012). Screening for distress and unmet needs in patients with cancer: review and recommendations. J Clin Oncol.

[71] Nir Z, Zolotogorsky Z, Sugarman H (2004). Structured nursing intervention versus routine rehabilitation after stroke. Am J Phys Med Rehabil.

[72] Li J, Wang L, Chao B, Liu Y (2015). Prevalence of stroke in China: an epidemiological study based on the National Stroke Screening Survey. Lancet..

