# Supplementary material for: Associations of psychological distress with positive psychological variables and activities of daily living among stroke patients: a cross-sectional study
Source: BMC Psychiatry. 2019 Dec 3;19:381. doi: 10.1186/s12888-019-2368-0 (PMC6888918; doi:10.1186/s12888-019-2368-0)
Supplement: Supplementary file 1 — Additional file 1. Mental health questionnaire for stroke patients. [file 12888_2019_2368_MOESM1_ESM.pdf]

No.: _____

Mental Health Questionnaire for Stroke Patients

Dear participant:

We thank you for your time.

We are investigating the status of your mental health and life quality, aiming to enhance the therapeutic effects and help in providing better treatments to stroke patients. Please finish this questionnaire truthfully. All information you provided will be treated in strictest confidence. Thanks for your participation.

Informed Consent

Research project: Survey on the mental health status of stroke patients.

I hereby agree and consent to join the survey. I volunteer for this project. I understand that I can withdraw from the project at any time. All my information is strictly confidential and can only be provided to the researchers of this project.

Signature: ___________

Data: _________

Please tick (√) your answers:

1. Age: ____; Gender: ① Male ② Female
2. Marital status: ① Married ② Single ③ Separated ④ Divorced ⑤ Widowed ⑥ Separated ⑦ Cohabiting
3. Education level: ① Primary school ② Middle school ③ High school ④ College
4. Residence type: ① Urban ② Rural
5. Chronic disease: ① Hypertension ② Heart disease ③ Diabetes ④ Hyperlipemia ⑤ Fatty liver ⑥Gout ⑦ Other Chronic disease:____ ⑨ No chronic disease
6. Monthly income (US dollars): ① ≤318.8 ② 318.9-797.0 ③ ≥797.1
7. Medical payments types: ① Basic medical insurance for urban employees ② Medical insurance for urban residents ③ New cooperative medical insurance ④ Medical insurance for government officials ⑤ Commercial medical insurance ⑥ Other medical insurance:____________ ⑦ No medical insurance

1 〖Barthel Index〗

Please tick (√) the scores that reflect the effect level on your daily life.

Activities	Scores	Standards	Activities	Scores	Standards
Bowels	0<br>5<br>10	Incontinent (or needs to be given enemas)<br>Occasional accident<br>Continent	Bladder	0<br>5<br>10	Incontinent, or catheterized and unable to manage alone<br>Occasional accident<br>Continent
Grooming	0<br>5	Needs to help with personal care<br>Independent face/hair/teeth/shaving (implements provided)	Bathing	0<br>5	Dependent<br>Independent (or in shower)
Toilet use	0<br>5<br>10	Dependent<br>Needs some help, but can do something alone<br>Independent (on and off, dressing, wiping)	Feeding	0<br>5<br>10	Unable<br>Needs help cutting, spreading butter, etc., or requires modified diet<br>Independent
Transfers(bed to chair and back)	0<br>5<br>10<br>15	Unable, no sitting balance<br>Major help (one or two people, physical), can sit<br>Minor help (verbal or physical)<br>Independent	Mobility(on level surfaces)	0<br>5<br>10<br>15	Immobile or < 50 yards<br>Wheelchair independent, including corners, > 50 yards<br>Walks with help of one person (verbal or physical) > 50 yards<br>Independent (but may use any aid; for example, stick) > 50 yards
Dressing	0<br>5<br>10	Dependent<br>Needs help but can do about half<br>Unaided independent (including buttons, zips, laces, etc.)	Stairs	0<br>5<br>10	Unable<br>Needs help<br>Independent

2 〖Perceived Social Support Scale (PSSS)〗

Please tick (√) your answers in the table based on your current conditions.

Items	Strongly disagree	Disagree	Slightly disagree	Neutral	Agree	Slightly agree	Strongly agree
1. There is a special person who is around when I am in need.	1	2	3	4	5	6	7
2. There is a special person with whom I can share my joys and sorrows.	1	2	3	4	5	6	7
3. My family really tries to help me.	1	2	3	4	5	6	7
4. I get the emotional help and support I need from my family.	1	2	3	4	5	6	7
5. I have a special person who is a real source of comfort to me.	1	2	3	4	5	6	7
6. My friend really try to help me.	1	2	3	4	5	6	7
7. I can count on my friends when things go wrong.	1	2	3	4	5	6	7
8. I can talk about my problems with my family.	1	2	3	4	5	6	7
9. I have friends with whom I can share my joys and sorrows.	1	2	3	4	5	6	7
10. There is a special person in my life who cares about my feelings.	1	2	3	4	5	6	7
11. My family is willing to help me make decisions.	1	2	3	4	5	6	7
12. I can talk about my problems with my friends.	1	2	3	4	5	6	7

3 〖Adult Hope Scale (AHS)〗

Please tick (√) your answers in the table based on your current conditions.

Items	Completely incorrect	Mostly incorrect	Mostly correct	Completely correct
1．I can think of many ways to get out of a jam.	1	2	3	4
2．I energetically pursue my goals.	1	2	3	4
3. I feel tired most of the time.	1	2	3	4
4．There are lots of ways around any problem.	1	2	3	4
5. I am easily downed in an argument.	1	2	3	4
6．I can think of many ways to get the things in life that are most important to me.	1	2	3	4
7．I worry about my health.	1	2	3	4
8．Even when others get discouraged, I know I can find a way to solve the problem.	1	2	3	4
9．My past experiences have prepared me well for my future.	1	2	3	4
10．I've been pretty successful in life.	1	2	3	4
11. I usually find myself worrying about something.	1	2	3	4
12．I meet the goals that I set for myself.	1	2	3	4

4 〖Wagnild and Young Resilience Scale-14 (RS-14)〗

Please tick (√) your answers in the table based on your current conditions.

Items	Strongly disagree	Disagree	Slightly disagree	Neutral	Agree	Slightly agree	Strongly agree
1. I usually manage one way or another	1	2	3	4	5	6	7
2. I feel proud that I have accomplished things in life	1	2	3	4	5	6	7
3. I usually take things in stride	1	2	3	4	5	6	7
4.I am friends with myself	1	2	3	4	5	6	7
5. I feel I can handle many things at a time	1	2	3	4	5	6	7
6. I am determined	1	2	3	4	5	6	7
7. I can get through difficult times because I have experienced difficulty before	1	2	3	4	5	6	7
8. Self-discipline is important	1	2	3	4	5	6	7
9. I keep interested in things	1	2	3	4	5	6	7
10. I can usually find something to laugh about	1	2	3	4	5	6	7
11. My belief in myself gets me through hard times	1	2	3	4	5	6	7
12. In an emergency, I am someone people can generally rely on	1	2	3	4	5	6	7
13. My life has meaning	1	2	3	4	5	6	7
14. When I am in a difficult situation, I can usually find my way out of it.	1	2	3	4	5	6	7

5 〖General Perceived Self-efficacy Scale（GSE）〗

Please tick (√) your answers in the table based on your current conditions.

Items	Not at all true	Hardly true	Moderately true	Exactly true
1．I can always manage to solve difficult problems if I try hard enough.	1	2	3	4
2．If someone opposes me, I can find the means and ways to get what I want.	1	2	3	4
3．It is easy for me to stick to my aims and accomplish my goals.	1	2	3	4
4．I am confident that I could deal efficiently with unexpected events.	1	2	3	4
5．Thanks to my resourcefulness, I know how to handle unforeseen situations.	1	2	3	4
6 .I can solve most problems if I invest the necessary effort.	1	2	3	4

7 . I can remain calm when facing difficulties because I can rely on my coping abilities.	1	2	3	4
8 . When I am confronted with a problem, I can usually find several solutions.	1	2	3	4
9 .If I am in trouble, I can usually think of a solution.	1	2	3	4
10 . I can usually handle whatever comes my way.	1	2	3	4

6 〖Center for Epidemiologic Studies Depression Scale (CES-D) and Zung self-rating anxiety scale (SAS)〗

Note: A little of the time: The condition occurred less than 1 day within the last 1 week. Some of the time: The condition occurred at least 1 to 2 days within the last 1 week. Good part of the time: The condition occurred 3 to 4 days within the last 1 week. Most of the time: The condition occurred 5 to 7 days within the last 1 week.

Items	A little of the time	Some of the time	Good part of the time	Most of the time
1. I was bothered by things that usually don't bother me.	0	1	2	3
2. I did not feel like eating; my appetite was poor.	0	1	2	3
3. I felt that I could not shake off the blues even with help from my family or friends.	0	1	2	3
4. I felt I was just as good as other people.	0	1	2	3
5. I had trouble keeping my mind on what I was doing.	0	1	2	3
6. I felt depressed.	0	1	2	3
7. I felt that everything I did was an effort.	0	1	2	3
8. I felt hopeful about the future.	0	1	2	3
9. I thought my life had been a failure.	0	1	2	3
10. I felt fearful.	0	1	2	3
11. My sleep was restless.	0	1	2	3
12. I was happy.	0	1	2	3
13. I talked less than usual.	0	1	2	3
14. I felt lonely.	0	1	2	3
15. People were unfriendly.	0	1	2	3
16. I enjoyed life.	0	1	2	3
17. I had crying spells.	0	1	2	3
18. I felt sad.	0	1	2	3
19. I felt that people disliked me.	0	1	2	3
20. I could not get going.	0	1	2	3
21. I feel more nervous and anxious than usual.	0	1	2	3
22. I feel afraid for no reason at all.	0	1	2	3
23. I get upset easily or feel panicky.	0	1	2	3
24. I feel like I'm falling apart and going to pieces.	0	1	2	3
25. I feel that everything is all right and nothing bad will happen.	0	1	2	3
26. My arms and legs shake and tremble.	0	1	2	3
27. I am bothered by headaches neck and back pain.	0	1	2	3
28. I feel weak and get tired easily.	0	1	2	3

29. I feel calm and can sit still easily.	0	1	2	3
30. I can feel my heart beating fast.	0	1	2	3
31. I am bothered by dizzy spells.	0	1	2	3
32. I have fainting spells or feel like it.	0	1	2	3
33. I can breathe in and out easily.	0	1	2	3
34. I get numbness and tingling in my fingers and toes.	0	1	2	3
35. I am bothered by stomach aches or indigestion.	0	1	2	3
36. I have to empty my bladder often.	0	1	2	3
37. My hands are usually dry and warm.	0	1	2	3
38. My face gets hot and blushes.	0	1	2	3
39. I fall asleep easily and get a good night's rest.	0	1	2	3
40. I have nightmares.	0	1	2	3

This is the end of this questionnaire. Thanks for your participation.

Researcher's signature: ___________
